# Supplementary material for: Joint Application of Lactobacillus plantarum and Bacillus subtilis Improves Growth Performance, Immune Function and Intestinal Integrity in Weaned Piglets
Source: Vet Sci. 2022 Nov 30;9(12):668. doi: 10.3390/vetsci9120668 (PMC9781797; doi:10.3390/vetsci9120668)
Supplement: Supplementary file 1 [file vetsci-09-00668-s001.zip › vetsci-1962359-supplementary.pdf]

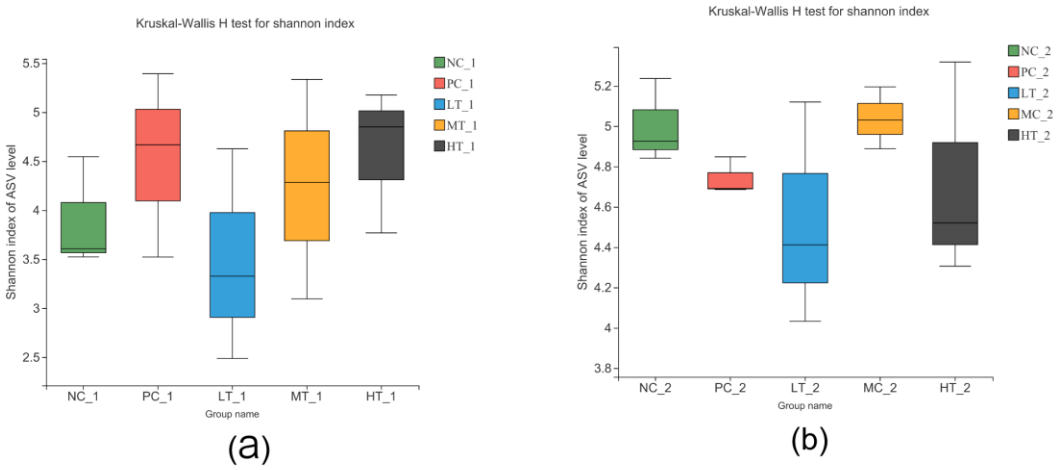

Supplementary Figure S1. The α- diversity of the microbial community in the jejunum and colonic digesta of weaned pigs. (**a**) Box plots showing microbial α-diversity (Shannon Index) in the jejunal digesta. (**b**) Box plots showing microbial α-diversity (Shannon Index) in the colonic digesta. NC, a control diet; PC, NC + 150 ppm mucilage sulfate; LT: 1kg/t joint application of *Lactobacillus Plantarum* and *Bacillus subtilis*; MT: 1.5 kg/t joint application of *Lactobacillus Plantarum* and *Bacillus subtilis*; HT: 2 kg/t joint application of *Lactobacillus Plantarum* and *Bacillus subtilis*.

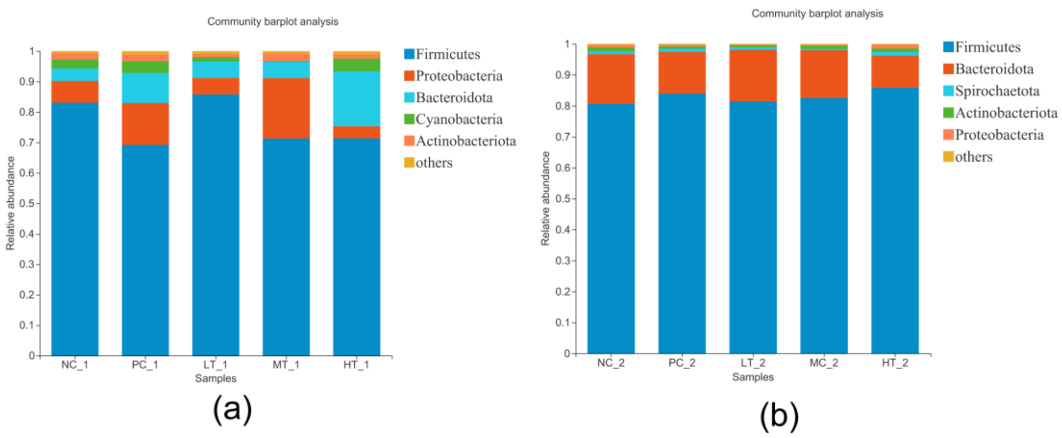

Supplementary Figure S2. The community bar plot analysis in the jejunum and colonic digesta of weaned pigs at the phylum level. (**a**) Characterization of jejunal microbial communities at the phylum level. (**b**) Characterization of colonic microbial communities at the genus level. NC, a control diet; PC, NC + 150 ppm mucilage sulfate; LT: 1kg/t joint application of *Lactobacillus Plantarum* and *Bacillus subtilis*; MT: 1.5 kg/t joint application of *Lactobacillus Plantarum* and *Bacillus subtilis*; HT: 2 kg/t joint application of *Lactobacillus Plantarum* and *Bacillus subtilis*.
